# A Participatory Survey to Investigate the Long-Term Effectiveness of Adult Psychiatric Services (PSILEAPS): Baseline Data and Recruitment Experiences

**DOI:** 10.1007/s10597-025-01462-z

**Published:** 2025-03-11

**Authors:** Tomi Bergström, Tapio Gauffin, Teija Hietasaari, Nina Koivuniemi, Anne Leinonen, Enni Öfverberg, Reetta Viitakoski, Timo Haaraniemi, Katja Partanen, Katariina Rantamaa, Vilhelmiina Yrjänheikki, Tiina Jauhiainen, Annika Olli, Jouko Miettunen, Jouni Petäjäniemi

**Affiliations:** 1 Department of Psychology, University of Jyväskylä, Jyväskylä, Finland; 2 Faculty of Medicine, University of Helsinki, Helsinki, Finland; 3 Department of Psychiatry, Wellbeing Services County of Lapland, Kemi, Finland; 4 Research Unit of Population Health, University of Oulu, Oulu, Finland; 5 Medical Research Center Oulu, Oulu University Hospital and University of Oulu, Oulu, Finland

**Keywords:** Participatory research, Adult psychiatric services, Mental health services, Recruitment experiences, Exploratory cohort study

## Abstract

In clinical psychology and psychiatry, traditional hypothesis testing is challenging due to the subjective and contextual nature of the studied phenomenon. To address this, more exploratory and participatory research is needed. This paper reports recruitment experiences and baseline data of a prospective exploratory cohort study with participatory elements, initiated in mental health services in one Finnish region. The primary aims, design, and survey for data collection were developed through community meetings involving local mental health workers, peer experts, and service users. Over 2 weeks, all mental health service users, their care teams, and social network members were asked to share their views on the reasons for needing services and what aspects of treatment have been or could be helpful or unhelpful. Descriptive statistics summarized baseline data, and simple thematic analysis examined field notes on supporting and hindering aspects of the study design. A total of 117 service users, 54 care team members, and 34 social network members participated, with a service user attrition rate of 40–50%. The study achieved 79% of the target sample size. Women and participants with mood disorder diagnoses and long-term service usage were overrepresented. Findings suggest that integrating participatory research into Finnish public mental health services would require additional resources. Despite its limitations, the collected data will facilitate exploratory research into real-life mental health treatment processes from various perspectives.

## Introduction

The World Health Organization (WHO) (2021) and the United Nations (2019) have raised concerns about the current state of global mental healthcare. Despite efforts, there has been no significant improvement in long-term treatment outcomes, and many countries have seen a rise in mental health disabilities. Services are struggling to provide adequate personalized treatment and support for those suffering from mental health difficulties (WHO, 2021). In response, the WHO (2021) has called for a shift in global mental health treatment and research practices from reductionistic models to more holistic approaches.

While dominant medical models have generated valuable information to support medical decision-making, their application in practice can sometimes overlook critical existential factors in mental healthcare. These factors include long-term social functionality, personal causal beliefs regarding mental distress, service users’ treatment preferences, and the real-world effectiveness of mental health interventions (Bergström, 2023; van Os et al., 2019). The holistic approaches and recovery movement have advocated for greater autonomy, dignity, and social inclusion beyond mere symptom reduction. Aligned with this, there has been an increasing emphasis on the involvement of service users and other stakeholders also in mental health research (Case et al., 2014).

More broadly, the field of psychological sciences has recognized issues with conventional, reductionistic approaches to hypothesis testing, stemming from validity and conceptual challenges related to the subjective and contextual nature of the studied phenomenon (Scheel et al., 2021). Similarly, in psychiatry, there is a need for more exploratory research activities to provide the necessary inputs for informative hypothesis testing (Bergström, 2023).

Participatory research designs provide a promising approach for conducting exploratory research in psychiatry. These designs engage clinicians, peer experts, and service users to collaborate with academics from problem identification through to the dissemination of results (Duea et al., 2022). This ensures that research questions, methodologies, and interpretations are more closely aligned with lived experiences and real-world clinical practice. In other words, this approach helps address the limitations of conventional methods by promoting more holistic, contextually grounded, and ecologically valid research in psychiatry. Beyond generating non-confirmatory scientific insights for the development of new, more precise hypotheses, participatory research also plays a role in shaping research-based mental health services that are more responsive to the needs of service users and practitioners.

Here, we focus on an effort to integrate a participatory and exploratory research elements into public mental health care services—specifically, a prospective exploratory cohort study titled “A Participatory Survey to Investigate the Long-Term Effectiveness of Adult Psychiatric Services” (PSILEAPS) (Bergström et al., 2022a). The first aim of PSILEAPS is to produce ecologically valid information on real-world mental health treatment and the factors associated with different treatment outcomes in one Finnish mental health service catchment area. This information can be used to generate new hypotheses for testing in more confirmatory research designs. The second aim is to integrate participatory research elements into everyday services and to evaluate whether this can support bottom-up service development. In this paper, we report baseline data of the project and initial experiences from the data collection.

## Materials and Methods

### Western Lapland Catchment Area

The Western Lapland (WL) catchment area comprises the southwestern parts of Finnish Western Lapland, with a population of 62000 in 2023. Prior to 2023, most of the adult mental health services in this area operated under the Länsi-Pohja Hospital District. This included two adult outpatient clinics in the towns of Kemi and Tornio, a secondary-level emergency outpatient clinic serving the entire region (formerly Keropudas outpatient clinic), and a psychiatric ward with 20 beds at Kemi Central Hospital, which was relocated from the old Keropudas Psychiatric Hospital in 2022.

The area has a history of developing social network-oriented, open dialogue-based psychiatric services (Seikkula & Olson, 2003). Although there has been some variation in and reduction of resources dedicated to open dialogue-based training, some of the structural elements of this approach remained at the time of recruitment. These included a centralized service intake, operating 24/7 from a single contact number, and no referrals being required to initiate mental health treatment.

### Design

The PSILEAPS study utilizes a participatory research elements that involves various community stakeholders, including mental health workers, peer experts, service users, and their family members. The project began in 2019 with open community meetings, bringing together local mental health workers and peer experts from Western Lapland (WL) mental health services. Meetings were open to all stakeholders through invitations sent via email and regular team meetings. To ensure accessibility, two meetings were held in each town, with 15–25 workers attending, representing the majority of outpatient staff. Despite the open invitation, hospital ward staff did not regularly participate in the community meetings. However, at the time, the principal investigator primarily worked on the ward and was involved in both the research project and regular team meetings, where research ideas and discussions were integrated. At the time, 2–4 peer experts were actively working in the services, and all of them attended at least one meeting, with many participating in multiple sessions. During these meetings, the initial project goals were established through open dialogue facilitated by the first author. Additionally, three structured questionnaires were drafted to gather input from care team members, service users, and their social network members.

In 2020, the questionnaire was distributed to all staff members in WL, and feedback was solicited regarding its relevance to clinical work. Following data collection, additional community meetings were held to finalize the questionnaire, incorporating insights from the pilot phase and feedback from staff members. The questionnaire was also reviewed with service users during regular hospital meetings at the local psychiatric hospital, where all patients currently in treatment gathered, to ensure that the questionnaire was relevant and clear from their perspective. Based on these discussions, certain questions were refined and clarified to improve their clarity and relevance.

The final questionnaire is structured around the biopsychosocial model of mental health problems, addressing the need for mental health services, available treatment approaches, and inquiries about service users’ current mental well-being, functionality, and changes in mental health and social functioning over the preceding month.

The first pilot of the participatory survey took place in 2020 at the Keropudas hospital’s outpatient clinic (Bergström et al., 2022b), involving responses from 11 care team members, 12 service users, and their social network members. The pilot demonstrated the feasibility of integrating participatory research into clinical practice, although some care team members noted time constraints, particularly when they had prepared specific treatment plans before outpatient meetings. Service users and their social network members found the questionnaire meaningful and beneficial, with no reported adverse effects. The pilot also highlighted challenges associated with remote meetings during the COVID-19 pandemic, leading to the postponement of the full-scale study from 2021 to 2022.

### Questionnaire

The first part of the questionnaire is structured around the biopsychosocial model of mental health problems. It includes three questions, each accompanied by examples, aimed at exploring the reasons why a particular service user may or may not require mental health services. The second part consists of three questions addressing how the service user’s situation should be approached. The third part includes a list of all the service and treatment approaches currently available in WL mental health services. Each care team member, service user, and their social network members indicated which of these treatment methods and approaches have been or may be helpful in a given treatment process. The final part of the questionnaire contains inquiries regarding the service user’s current mental well-being, functionality, and any improvement or decline in their mental health and social functioning over the last month. A more detailed description and content of the questionnaire can be found in the research protocol (Bergström et al., 2022a).

### Data Collection

The full-scale study was initiated in spring 2022 by first administering the questionnaire in Tornio and Keropudas outpatient clinics. All staff members were instructed to invite all service users within a two-week timeframe. Invitation letters and questionnaires were also made available in the waiting areas. Recruitment at the Kemi outpatient clinic and psychiatric ward followed a similar process in autumn 2022.

Background information regarding demographic and clinical characteristics, including both somatic and psychiatric morbidity prior to participation, was gathered directly from service users and from case note files, based on their informed consent. Psychiatric symptoms and their severity were evaluated by care team members using the Brief Psychiatric Rating Scale (BPRS). All participants were also asked to provide informed consent for a register-based follow-up, where demographic and clinical information will be gathered from national registers over a five-year period from the time of participation.

Service users who agreed to participate, along with their chosen social network members and care team members, each completed a questionnaire. Care team members collected background information from each treatment process and delivered these materials to the principal investigator for data preservation, combination, and pseudonymization. All service users had the opportunity to review the responses in meetings with their care team members and/or with the principal investigator. They were also given the option to return the questionnaire without their care team members seeing their responses.

During the first data collection period, 89 patients provided informed consent to participate in the study. Based on general service caseload data, it was estimated that 50% of potential candidates in Kemi and 40% in Tornio returned the questionnaire. Since the targeted sample size was not reached during the initial data collection period, recruitment was resumed in a similar manner during autumn 2023. Following this second recruitment period, a total of 117 participants were recruited, comprising 79% of the targeted sample size. Given that the response rate during the second period was less than 30%, and in light of significant national healthcare system reforms in 2023, no further recruitment periods were held.

### Experiences on Data Collection

The principal investigator (TB), who worked as a clinical psychologist at the research sites during data collection, also coordinated recruitment efforts. Throughout the process, he drafted field notes from each unit’s weekly team meetings before, during, and after the recruitment periods to document experiences and feedback on data collection. All participants were also given the opportunity to provide feedback on the study design, either directly in face-to-face meetings or through research forms.

### Analysis

Non-confirmatory exploratory approaches were used to analyze the baseline data, focusing primarily on descriptive information about the sample characteristics and research experiences. Electronic databases on service usage were employed to conduct a basic attrition analysis by comparing the available demographic and clinical characteristics of participants with those of the entire service user population in the catchment area in 2022.

Field notes were analyzed through simple thematic analysis to identify major and sub-themes related to the recruitment experiences from different stakeholder perspectives. Both the field notes and the actual outcome data were discussed and reviewed with care team members during weekly team and additional community meetings.

### Ethical Considerations

The Northern Ostrobothnia Hospital District ethical committee approved the questionnaire and the participatory study design in 2020. Participation in the study was entirely voluntary and based on informed consent. It was explicitly communicated, both in writing and verbally, that declining to participate would not affect the treatment of patients or family members, nor would it impact the work conditions of care team members.

All participants had the option to submit their responses and feedback in a sealed envelope directly to the principal investigator, who was not involved in their treatment, ensuring that care team members would not see their responses. Additionally, all participants were given the opportunity to discuss their experiences and provide feedback with care team members, supervisors, or privately with the principal investigator.

## Results

### Sample Characteristics

#### Service Users

Baseline information is summarized in Table 1. A total of 117 service users participated in the study, with a majority being female (74%). The average age of participants was 38 years (Standard Deviation (SD): 15 years). The most common primary diagnoses were mood and anxiety disorders. Symptom severity, as evaluated by the Brief Psychiatric Rating Scale (BPRS) at the time of participation, indicated mild to moderate symptoms on average.

**Table 1:** Demographical and clinical background of service users

	Total sample	All service users in catchment area in year 2022
	n = 117	n = 3643
Gender, Female	87 (74%)	2197 (62%)
Age (mean (sd))	38 (15)	40 (16)
In relationship, yes	64 (55%)	NA
Children(s), yes	52 (44%)	NA
Highest education		NA
Lower secondary	19 (16%)	
Vocational upper secondary	64 (55%)	
General upper secondary	17 (15%)	
University of applied sciences	13 (11%)	
University	4 (3%)	
Unemployed	25 (21%)	NA
Disability allowances	46 (39%)	NA
Last primary diagnosis		
F1	4 (3%)	171 (5%)
F2	9 (8%)	263 (7%)
F3	53 (45%)	1042 (29%)
F4	26 (22%)	1011 (28%)
F5	1 (1%)	170 (5%)
F6	5 (4%)	177 (5%)
F7	0	35 (1%)
F8	2 (2%)	103 (3%)
F9	8 (7%)	238 (7%)
No F-diagnoses	9 (8%)	433 (12%)
BPRS [mean (sd)]	36 (11)	NA
Start of the treatment		
Within days	34 (29%)	
Within weeks	76 (65%)	
Within months	6 (5%)	
Within year	1 (1%)	
Lengh of the treatment		NA
Weeks	11 (9%)	
Months	30 (26%)	
Years	66 (56%)	
Decades	10 (8%)	

Most respondents had received some form of mental health treatment for many years prior to participation. Around half were referred to services after their initial treatment contact, and a similar proportion had received hospital treatment at some point before joining the study. The most common meeting frequency at the time of participation was 1–2 meetings per month. The majority of participants reported receiving help within weeks of their first contact with services.

According to a crude attrition analysis, there was an overrepresentation of female participants and those with mood disorder diagnoses compared to the total service user population in the catchment area during 2022.

#### Care Team and Family Members

A total of 54 workers participated in the study. During the initial data collection period, all workers in outpatient clinics who were on duty participated, while the participation rate was significantly lower among psychiatric ward staff. Despite direct recruitment efforts during hospital stays and encouragement for hospital staff to complete the questionnaires, service users may have been reluctant to respond and return them while hospitalized. Since staff member responses depended on service users completing and submitting their questionnaires, this may have contributed to participant attrition, resulting in lower response rates among staff members. Additionally, given the generally short inpatient stays (averagely less than a two weeks), outpatient staff likely coordinated much of the recruitment process.

In line with the general distribution of staff, most care team members were nurses, with an average of 14 years of work experience. Twenty-seven staff members had completed a local three-year Open Dialogue training, and four were also trained for other form of psychotherapists (Table 2).

**Table 2:** Care team members

	n = 54
Unit	
Tornio outpatient clinic	22 (42%)
Kemi outpatient clinic	18 (34%)
Psychiatric ward/emergency clinic	14 (26%)
Profession	
Nurse	38 (70%)
Psychologist	8 (15%)
Social worker	4 (7%)
Occupational therapist	2 (4%)
Doctor	2 (4%)
Working experience in years [mean (sd)]	14 (11)
Further education [mean (sd)]	4 (4)
> 3 year open dialogue training	27 (50%)
Other > 3 year psychotherapist training	4 (7%)

Thirty family members participated in the study. Their reported relationships to the service users were as follows: 11 (37%) mothers, 3 (10%) fathers, 11 (37%) spouses, 3 (10%) daughters, 1 (3%) support person, and 1 (3%) friend.

### Experiences in Data Collection

During and after recruitment, the principal investigator attended two team meetings in each unit, where staff members were encouraged to share their experiences with the research process. Based on the simple thematic analysis of field notes, nine subthemes were identified regarding recruitment experiences, which were categorized into two major thematic groups: experiences that either supported or hindered the recruitment and data collection process.

#### Supportive Experiences

During team meetings, staff members highlighted several factors that supported the recruitment process. Across all units, there was a consensus on the importance of obtaining direct feedback from service users at every phase of the treatment, which motivated their participation. Staff reported that service users viewed the research invitation positively, feeling heard and understood. The accessibility and relevance of the questionnaire were also well-received.

#### Hindering Experiences

Several challenges were identified that hindered recruitment. Care team members faced difficulties with time management, as the recruitment process, particularly the completion of questionnaires and background information forms for each participant, was time-consuming. While the staff appreciated having research materials prepared for them, the volume of forms—including consent forms and various questionnaires—felt overwhelming and confusing, especially before recruitment began. Some care team members noted that these challenges eased as data collection progressed, but the amount of paperwork and time required likely impacted their motivation to recruit participants.

In the later phases of recruitment, the response rate was lower than during the initial round. According to staff members, one potential reason for this was organizational changes, including shifts in staff during the study period. As a result, there were fewer staff members familiar with the research and its primary purpose, which may have affected data collection. Additionally, staff noted that treatment intensity had decreased, making it even more challenging to follow up with and remind participants who had not returned their forms**.** Some participants from the initial phase remained the treatment and this also affected the total participation rate in the second phase (Table 3).

**Table 3:** Thematic content on the field notes from care team member team meetings prior, during and after recruitment periods

	Experiences supporting the recruitment
Staff related	Importance of feedback
	Prepared materials
Service user related	Feeling heard
	The easy accessibility
	Experiences hindering the recruitment
Staff related	Time management
	Paper work
	Organizational changes
Service user related	Forgetfulness
	Secondary use of health data

#### Service User Feedback

None of the service users provided direct feedback to the principal investigator, and no negative feedback regarding the study design was reported in the research forms. Therefore, service user experiences are based solely on those reported by their care team members. Although many service users and their family members, as reported by staff in team meetings, valued the opportunity to provide direct feedback on their treatment and found the questions accessible, many did not return their informed consent forms. According to care team members, the most common reason was that service users simply forgot to complete the forms, despite reminder. Another reason mentioned was concerns about the secondary use of health data, which required informed consent. Although the use of healthcare data for research purposes was stated, many participants were worried about how their information would be used.

## Discussion

In this paper, we described baseline information and shared our recruitment experiences in a study aimed at gathering insights from stakeholders on everyday mental health services. Over 2-week timeframes, we invited all service users and their care team members within a specific geographical catchment area of Finnish mental health services to provide feedback on several key aspects: their reasons for using mental health services, their perspectives on care organization, the effectiveness of available treatment modalities, whether they received the desired help, and how their well-being and functional abilities had evolved over the past month. With participants' consent, their chosen social network members also provided similar information regarding the service users' situations.

Approximately half of the invited service users responded to the questionnaire. Attrition analysis suggests that the sample may lack data from male service users, while female service users were overrepresented. Clinically, the participants had been receiving services for a significant period, and compared to the overall regional population, there were fewer participants without an F-diagnosis. This indicates a possible underrepresentation of those with shorter-term or crisis-oriented treatment, which may affect the generalizability of the PSILEAPS project’s findings to the local service user population. Nevertheless, the age, gender, and diagnostic distributions in the sample align with the overall patterns in mental health services, where mood and anxiety disorders are most common, and the majority of service users are women.

Experiences from design demonstrates that integrating systematic data collection directly from stakeholders into everyday services is possible at some level, but continuous data collection may not be feasible with our current design due to the burdensome nature of recruitment and the process of obtaining consent for the secondary use of healthcare data. An alternative approach could involve allocating additional resources for recruitment, data management, and collection, but this was not feasible given the current resources and funding in Finnish public healthcare services. Additionally, the time required to complete the questionnaires was often too demanding for many stakeholders, which also likely contributed to the response rates among service users. Despite efforts to adopt a participatory approach in drafting the questionnaire to capture meaningful aspects, it is possible that our design failed to address certain group-specific factors that may have been more relevant and important to them, potentially affecting their motivation to participate.

These challenges mirror those encountered in previous research efforts aiming to include participatory elements. A review of participatory approaches in mental health identifies several key obstacles, including the need for continuous methodological adjustments, unclear role boundaries between researchers and community members, difficulties in engaging marginalized populations due to stigma and trust issues, barriers in data collection, and challenges in accurately measuring intervention outcomes, as traditional research metrics may not fully capture the holistic impact of community-based approaches (Stacciarini et al., 2011). Effective participatory approach requires training and ongoing support for stakeholders, making it more time-intensive than traditional research (Case et al., 2014). Institutional constraints, such as funding cycles and rigid research timelines, may not align with the evolving nature of this type of projects (Rodriguez Espinosa & Verney, 2021).

In our study, the strategy we employed may not have been entirely effective in mitigating power dynamics and thereby fostering a more inclusive participatory framework. The responsibility for coordinating the study largely rested with a single researcher who worked within the organization. Although participation in the planning and conducting research was encouraged and open at all stages for all stakeholders, multiple factors may have made it difficult for individuals to attend and/or share their experiences. Such challenges could be addressed by establishing clearer research structures and defining associated roles, as well as by providing appropriate compensation for contributions. However, due to resource constraints, this was not feasible within the scope of our design.

### Strengths and Limitations

A key strength of the study design was its attempt to collaborative approach, involving stakeholders in planning and conducting the study. A limitation is that the data may not be generalizable or directly comparable with other catchment areas. In the future, generalizability can be enhanced by replicating a similar procedure. However, we observed several limitations affecting the feasibility of this process and its ability to achieve its primary purpose—namely, to simultaneously strengthen participatory aspects and maintain a continuous exploratory research design for bottom-up service development.

First, the study was conducted without additional resources, allowing us to test how this type of design could be integrated into everyday clinical practice. While this approach provided valuable insights, it also led to less systematic data collection, potentially leaving certain factors influencing the design and recruitment process undetected. Importantly, we were unable to obtain direct feedback from service users who declined to participate, which may have introduced selection bias into the upcoming analyses. It is for example possible that those who found research irrelevant or experienced it as a negative, did not returned their forms.

All stakeholders were actively encouraged to provide feedback, either directly to the principal investigator or indirectly through research forms. However, various factors—some of which may have been unidentifiable or beyond reach—may have contributed to the limited feedback received. For example, due to limited resources, we were unable to systematically collect individual feedback from both service users and staff members on their experiences with the research and its design.

The principal investigator’s close involvement in each unit as a clinical psychologist may have also influenced how feedback was given. However, this involvement also provided deeper insight into the actual recruitment and research experiences compared to an external reviewer or feedback forms. Nevertheless, the use and thematic analysis of field note data were largely based on subjective interpretations shared during team meetings, and both the feedback received and its interpretation may have been shaped by the principal investigator’s role.

## Conclusion

Baseline analysis of PSILEAPS project, which involved stakeholders in planning and data collection within everyday mental health practice, demonstrates that integrating such an approach into Finnish public mental health services could be possible to some extent with our design. However, more systematic and ongoing implementation, along with an increased rate of participation, would require additional resources. These resources would enable stakeholders, including staff members, service users, and their family members, to work more systematically and potentially lead the project, which would positively impact the primary aim of the participatory design. Additional resources would also allow for more systematic and individual forums to discuss the research experiences and findings, which were now mainly addressed in everyday team meetings and service user community meetings at the psychiatric hospital.

In summary, our experiences suggest that additional or rearrangement of resources are needed to effectively integrate ongoing exploratory research designs with participatory elements into service delivery. This would support the co-production of information needed to develop services and adequately compensate stakeholders for their efforts. In the future, services should explore whether such resources can be obtained through organizational restructuring of work tasks, third-party support, or external funding sources. It is also crucial to develop more flexible data collection methods and create forums for collaboratively interpreting findings while reducing power hierarchies.

Despite these limitations, 79% of the targeted sample size was reached, and the data collected will enable exploratory research on actual treatment processes from different perspectives. It is anticipated that this data will provide new insights into real-life mental health services.
